# Supplementary material for: Chronic Effects of Imidacloprid on Honey Bee Worker Development—Molecular Pathway Perspectives
Source: Int J Mol Sci. 2021 Oct 31;22(21):11835. doi: 10.3390/ijms222111835 (PMC8584158; doi:10.3390/ijms222111835)
Supplement: Supplementary file 1 [file ijms-22-11835-s001.zip › Figures S1 and S2.pptx]

## Slide 1
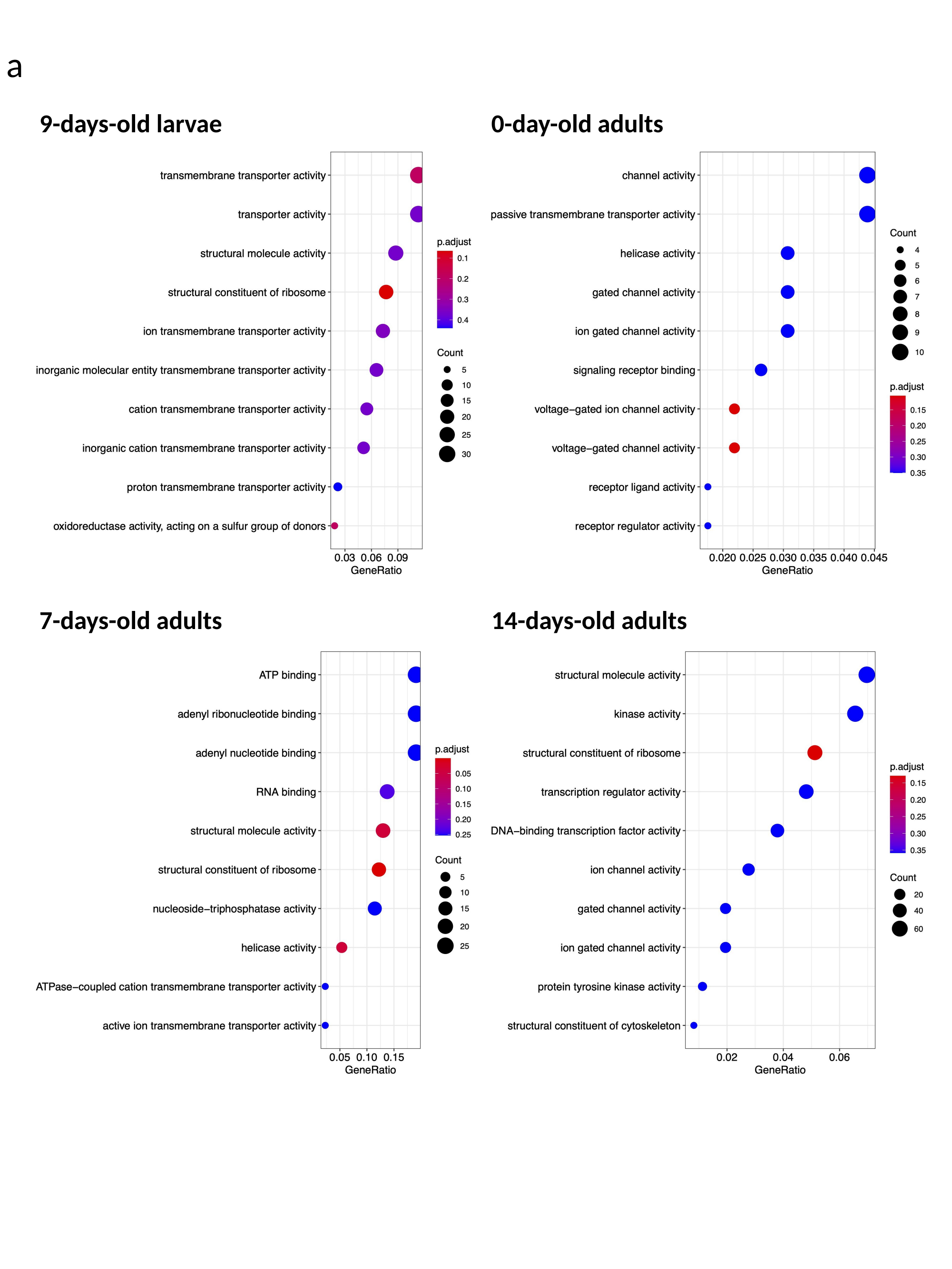

a
9-days-old larvae
0-day-old adults
7-days-old adults
14-days-old adults

## Slide 2
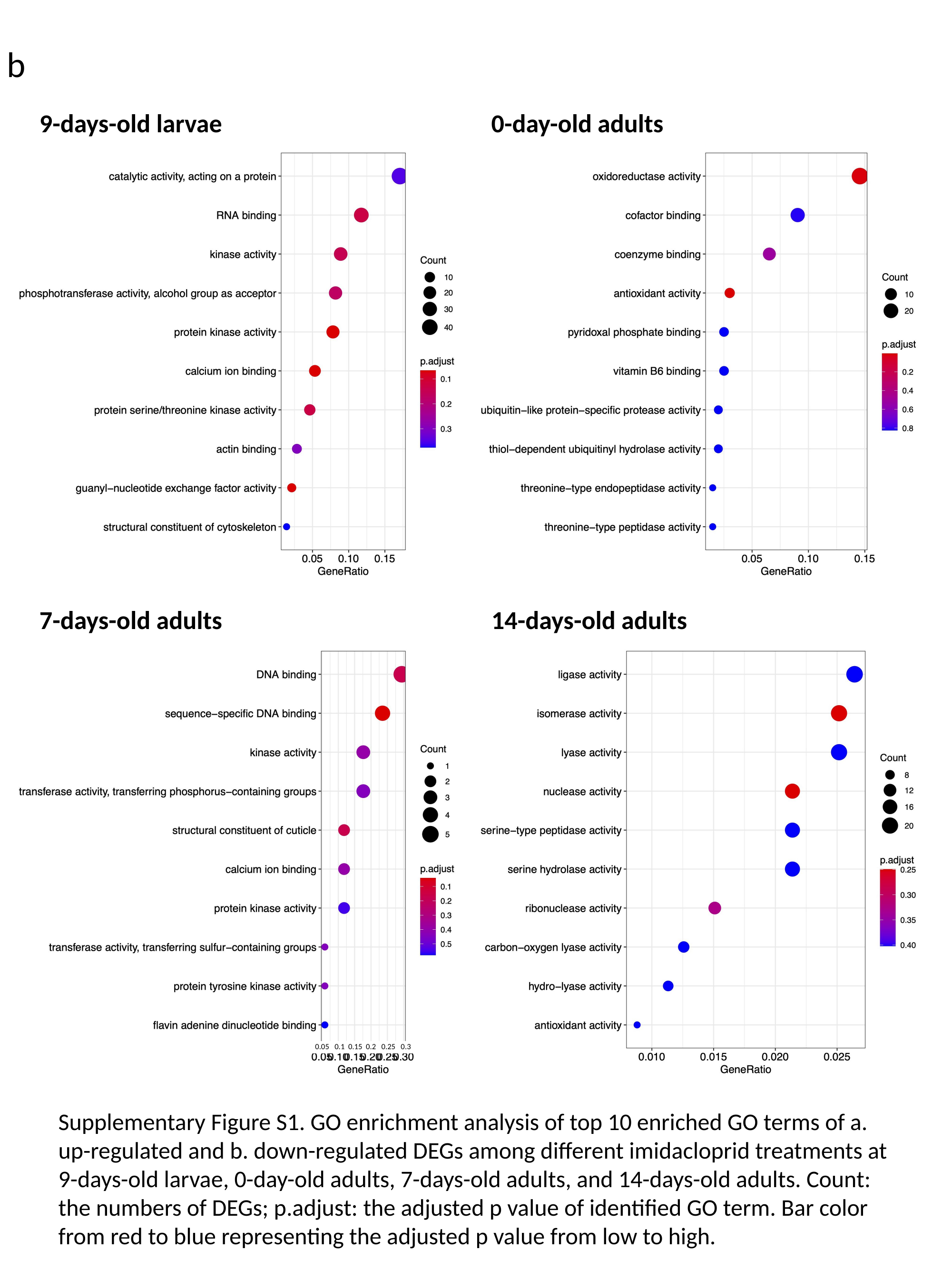

b
9-days-old larvae
0-day-old adults
7-days-old adults
14-days-old adults
0.05 0.1 0.15 0.2 0.25 0.3
Supplementary Figure S1. GO enrichment analysis of top 10 enriched GO terms of a. up-regulated and b. down-regulated DEGs among different imidacloprid treatments at 9-days-old larvae, 0-day-old adults, 7-days-old adults, and 14-days-old adults. Count: the numbers of DEGs; p.adjust: the adjusted p value of identified GO term. Bar color from red to blue representing the adjusted p value from low to high.

## Slide 3
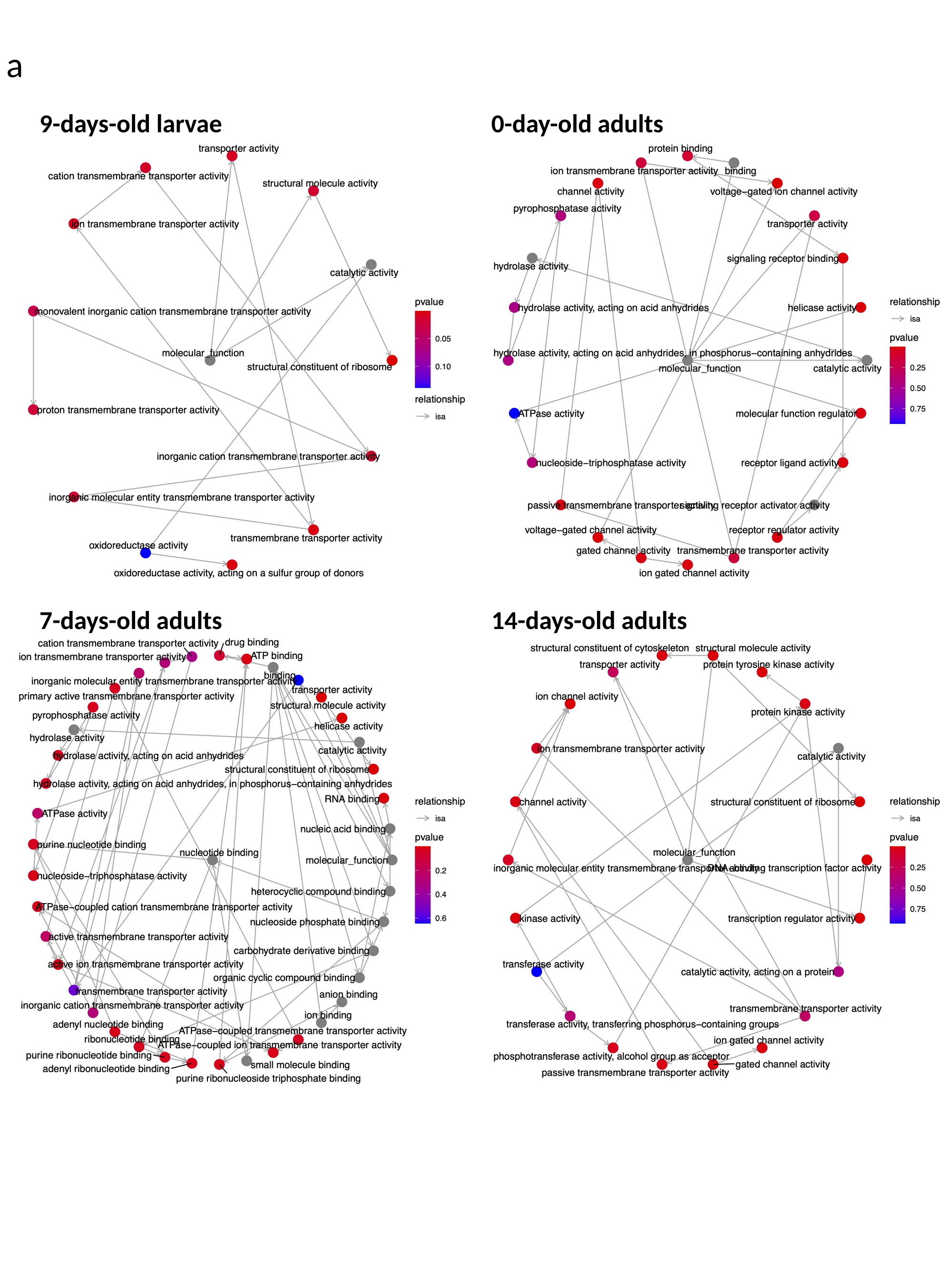

a
9-days-old larvae
0-day-old adults
7-days-old adults
14-days-old adults

## Slide 4
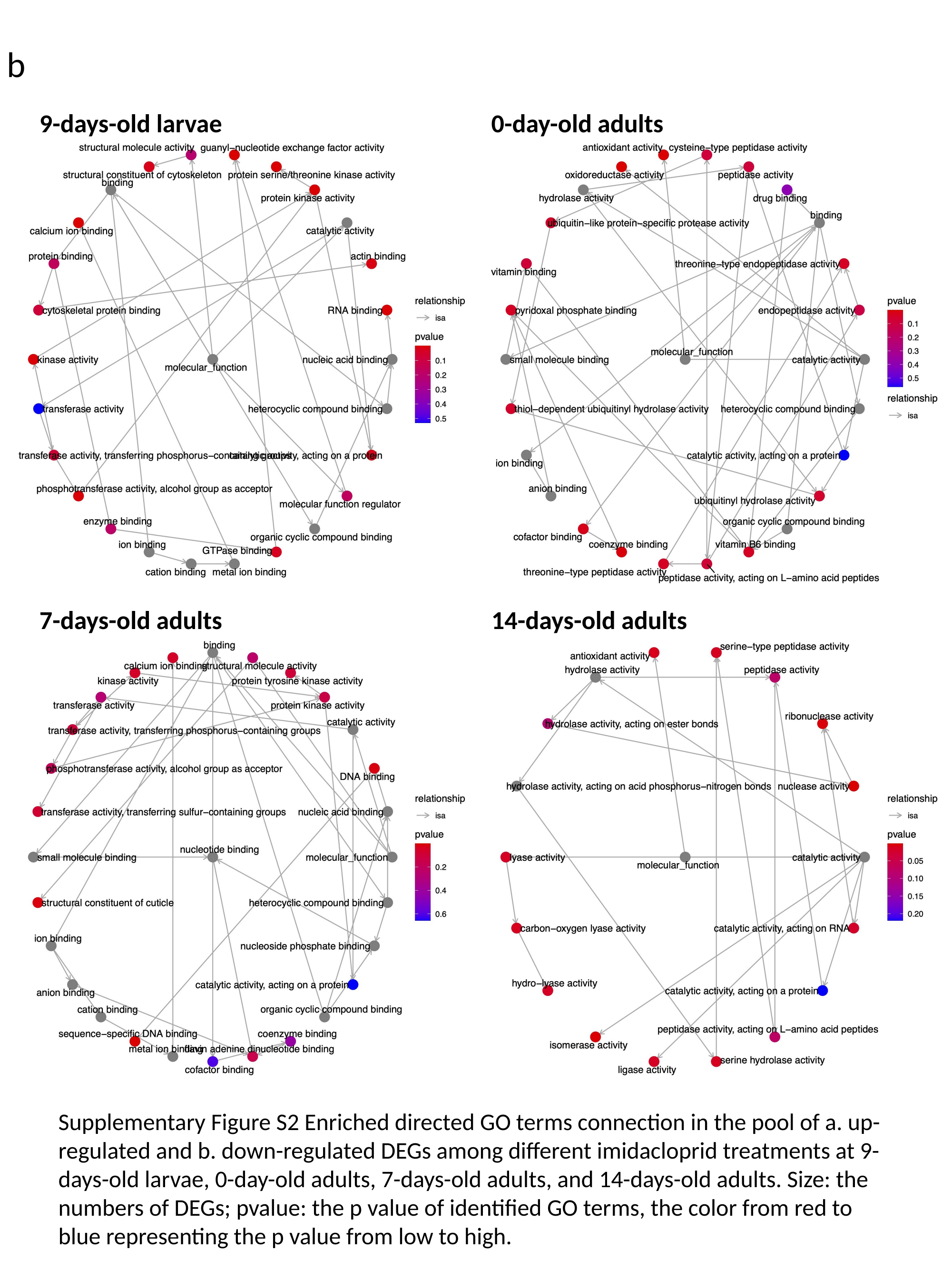

b
9-days-old larvae
0-day-old adults
7-days-old adults
14-days-old adults
Supplementary Figure S2 Enriched directed GO terms connection in the pool of a. up-regulated and b. down-regulated DEGs among different imidacloprid treatments at 9-days-old larvae, 0-day-old adults, 7-days-old adults, and 14-days-old adults. Size: the numbers of DEGs; pvalue: the p value of identified GO terms, the color from red to blue representing the p value from low to high.
